# Antimicrobial Activity of Cinnamaldehyde on *Streptococcus mutans* Biofilms

**DOI:** 10.3389/fmicb.2019.02241

**Published:** 2019-09-25

**Authors:** Zhiyan He, Zhengwei Huang, Wei Jiang, Wei Zhou

**Affiliations:** ^1^Laboratory of Oral Microbiota and Systemic Diseases, Shanghai Key Laboratory of Stomatology and Shanghai Research Institute of Stomatology, Shanghai Ninth People’s Hospital, College of Stomatology, Shanghai Jiao Tong University School of Medicine, Shanghai, China; ^2^National Clinical Research Center for Oral Diseases, Shanghai Key Laboratory of Stomatology and Shanghai Research Institute of Stomatology, Shanghai, China; ^3^Department of Endodontics, Shanghai Ninth People’s Hospital, College of Stomatology, Shanghai Jiao Tong University School of Medicine, Shanghai, China

**Keywords:** *Streptococcus mutans*, cinnamaldehyde, biofilm, antimicrobial activity, virulence, dental caries

## Abstract

*Streptococcus mutans* is considered the most relevant bacteria in the transition of non-pathogenic commensal oral microbiota to biofilms which contribute to the dental caries process. The present study aimed to evaluate the antimicrobial activity of a natural plant product, cinnamaldehyde against *S. mutans* biofilms. Minimum inhibitory concentrations (MIC), minimal bactericidal concentration (MBC), and growth curves were determined to assess its antimicrobial effect against planktonic *S. mutans*. The biofilm biomass and metabolism with different concentrations of cinnamaldehyde and different incubation time points were assessed using the crystal violet and MTT assays. The biofilms were visualized using confocal laser scanning microscopy (CLSM). Bacterial cell surface hydrophobicity, aggregation, acid production, and acid tolerance were evaluated after cinnamaldehyde treatment. The gene expression of virulence-related factors (*gtfB*, *gtfC*, *gtfD*, *gbpB*, *comDE*, *vicR*, *ciaH*, *ldh* and *relA*) was investigated by real-time PCR. The MIC and MBC of cinnamaldehyde against planktonic *S. mutans* were 1000 and 2000 μg/mL, respectively. The results showed that cinnamaldehyde can decrease biofilm biomass and metabolism at sub-MIC concentrations. CLSM images revealed that the biofilm-covered surface areas decreased with increasing concentrations of cinnamaldehyde. Cinnamaldehyde increased cell surface hydrophobicity, reduced *S. mutans* aggregation, inhibited acid production, and acid tolerance. Genes expressions in the biofilms were down-regulated in the presence of cinnamaldehyde. Therefore, our data demonstrated that cinnamaldehyde at sub-MIC level suppressed the microbial activity on *S. mutans* biofilm by modulating hydrophobicity, aggregation, acid production, acid tolerance, and virulence gene expression.

## Introduction

Dental caries is a disease of chronic progressive destruction that occurs as a result of dysbiosis among commensal and pathogenic bacteria. It leads to demineralization of the tooth surface within an ecosystem of high density and diversity, known as dental biofilm (plaque), with increased acid production from microbial action of bacteria after intake of dietary fermentable carbohydrates (Nyvad et al., 2013; Kaur et al., 2017; Al-Dulaijan et al., 2018). A biofilm (plaque) is an ecological environment formed on the surface by microbial community and is made of self-produced extracellular polymeric substances (EPS) matrix consisting of protein, polysaccharides, and nucleic acid (Liu and Yu, 2017; Ye et al., 2019). According to the ecological plaque hypothesis, both the related bacteria and ecological changes caused by other factors can interfere with the ecological balance between the host and microbes (Anderson et al., 2018).

*Streptococcus mutans*, a Gram-positive bacterium in oral cavity, causes dysbiosis in this symbiotic ecosystem, although it is not solely responsible for the disease progression. It is considered as the most relevant bacteria in the transition of non-pathogenic commensal oral microbiota to biofilms which contribute to the dental caries process (Martins et al., 2018). *S. mutans* has developed multiple mechanisms to colonize the tooth surface and form bacterial plaque biofilm (Ren et al., 2016; Hu et al., 2018b). The ability of this bacterium to produce organic acids through various carbohydrate metabolism processes (acidogenicity) and survive in low pH environment (aciduricity) are major virulence factors in biofilm and lead to the development to dental caries (Cai et al., 2017; Chakraborty and Burne, 2017). The biofilm phenotype is physiologically and functionally distinct from the planktonic bacteria. Bacteria in the biofilm exhibit reduced metabolic activity and physiology. The biofilm structure serves as a physical barrier which limits penetration of antimicrobial agents into the deep layers of biofilm. Thus, bacteria growing in a biofilm increase its tolerance to antibiotics and immune resistance to the host (Hu et al., 2018a; Kuang et al., 2018; Ong et al., 2018). Since reckless and continuous use of antibiotics has led to a rapid increase in antibiotic resistance to conventional therapies, there is an urgent need to develop novel antimicrobial agents in order to inhibit biofilm formation.

Natural plant products, mainly phytochemicals and their derivatives have been used as major sources of effective therapeutic agents throughout history and are considered as alternatives to antibacterial agents. Their advantages include relative inexpensiveness; abundant sources (such as fruits, seeds, and vegetables); low levels of cytotoxicity; high chemical diversity and biochemical specificity; and less prone toward developing resistance to antibiotics (Jeon et al., 2011; Abreu et al., 2016; Borges et al., 2016). Cinnamaldehyde, an α, β-unsaturated aromatic aldehyde, is a major component in Chinese cinnamon essential oil (Table 1; Ribeiro et al., 2018). It is widely used as a flavoring agent in the food, beverages, and perfume industries (Adams et al., 2004; Lee and Balick, 2005). Cinnamaldehyde has been reported to be effective against Gram-positive and Gram-negative bacterial biofilms, such as those formed by *Pseudomonas aeruginosa* and *Staphylococcus aureus* (Zhang et al., 2014; Topa et al., 2018). Therefore, in the present study, we investigated the antimicrobial activity of cinnamaldehyde on the biofilm formed by *S. mutans* at sub-MIC levels. This study may aid the development of a natural product as a novel therapeutic agent to counteract the virulence effect of *S. mutans*; thus, cinnamaldehyde has the potential to be used in treatment of dental caries.

**TABLE 1:** Chemical structure and molecular properties of cinnamaldehyde.

**Cinnamaldehyde**	
Chemical structure	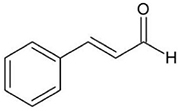
Molecular weight (g/mol)	132.16
logP (octanol-water partition coefficient)	2.48
n-ROTB (number of rotable bonds)	2
TPSA (topological polar surface area)	17.07
n-OH (number of hydrogel bond acceptors)	1

## Materials and Methods

### Bacterial Strain and Growth Condition

*Streptococcus mutans* UA159 strain and *Streptococcus sanguinis* SK36 strain were provided by Laboratory of Oral Microbiota and Systemic Diseases, Shanghai Ninth People’s Hospital, Shanghai Jiao Tong University School of Medicine. *S. mutans* and *S. sanguinis* were grown in Brain Heart Infusion Broth (BHI; Difco Laboratories, Sparks, MD, United States) at 37°C under anaerobic conditions (80% N_2_, 10% CO_2_, and 10% H_2_).

Determination of Minimum Inhibitory Concentration (MIC) and Minimum Bactericidal Concentration (MBC)

The MIC and MBC of cinnamaldehyde against planktonic *S. mutans* were determined by the reference protocol of the Clinical and Laboratory Standards Institute (2012) broth method. The cinnamaldehyde (Sigma-Aldrich) was diluted in 0.5% dimethyl sulfoxide (DMSO) and tested at final concentrations of 250–4000 μg/mL. Bacterial suspensions (5 × 10^5^ CFU/mL) were added and then incubated at 37°C for 24 h. To validate the methodology used in this study, we used a blank control (sterile culture medium, without cinnamaldehyde and suspensions of microorganisms) and a vehicle control (sterile culture medium with DMSO). The MIC was determined as the lowest drug concentration that inhibited visible bacterial growth. The MBC was defined as the lowest concentration that yielded no colony growth by subculturing on agar plates. Each experiment was performed with triplicate samples at each time point. The results correspond to three experiments independently.

### Growth Curve Assay

The overnight culture of *S. mutans* was used to anaerobically inoculate a fresh BHI culture with different concentrations of cinnamaldehyde (0, 62.5, 125, 250, 500, and 1000 μg/mL) at 37°C anaerobically for 24 h. Chlorhexidine (0.2%) was used as positive control. The optical density at 600 nm (OD_600__nm_) was measured by a spectrophotometer (UV1601, Shimadzu, Japan) every 3 h throughout incubation. Each experiment was performed with triplicate samples at each time point. The results correspond to three experiments independently.

### Crystal Violet Assay

Crystal violet (CV) assay was used to provide an overall assessment of biofilm biomass in a 96-well microtiter plate (Bedran et al., 2014). An overnight culture adjusting the OD_600__nm_ to 0.1 (10^8^ CFU/mL) was added to 180 μL of fresh BHI liquid medium supplemented with 0.2% sucrose in each flat-bottom well with different concentrations of cinnamaldehyde (0, 62.5, 125, 250, and 500 μg/mL). The dual-species biofilm was formed as previously described in detail (Guo et al., 2019). The positive control was set as chlorhexidine (0.2%). The plates were then incubated at 37°C for different times (4, 24 h) without agitation. After incubation, the growth medium was gently removed, washed three times with sterile phosphate-buffered saline (PBS) and replaced with 100 μL CV. The plates were incubated for 10 min at room temperature. The excess CV solution was removed, and wells were rinsed three times with PBS and the bound CV was dissolved by adding 100 μL 95% ethanol. The absorbance of the solution was measured at a wavelength of 550 nm by microplate reader. Each experiment was performed with triplicate samples at each time point. The results correspond to three experiments independently.

### MTT Assay

MTT [3-(4,5-dimethylthiazol-2-yl)-2,5-diphenyltetrazolium bromide] assay was used to assess the metabolic activities of viable biofilm cells (He et al., 2012). The biofilm was formed as described above. After incubation, the growth medium was gently removed, carefully washed three times with sterile PBS and replaced with 100 μL of MTT (5 mg/mL) for 3 h in a dark place. Next, the supernatant was discarded and 100 μL of lysing solution [10% (v/v) sodium dodecyl sulfate and 50% (v/v) dimethylformamide in distilled water] was added to dissolve the biofilm for 3 h at room temperature before reading the OD_590__nm_ values. Wells contained no cells were used as blank, and wells with chlorhexidine (0.2%) were used as positive controls. Each experiment was performed with triplicate samples at each time point. The results correspond to three experiments independently.

### CLSM Analysis

*Streptococcus mutans* biofilms were cultured on glass slides with different concentrations of cinnamaldehyde (0, 125, 250, and 500 μg/mL) for 24 h at 37°C. The biofilms formed on each sheet were washed three times with saline to remove unbound cells and stained for 30 min in the dark with L-7012 LIVE/DEAD BacLight TM bacterial cells (Molecular Probes Inc., Eugene, OR, United States) containing SYTO 9 dye and propidium iodide. A confocal laser scanning microscope (Leica TCS SP2, Leica microsystems, Germany) was used to record image stacks in five random locations. Five confocal data sets were recorded at 40 × magnification with a numerical aperture of 1.25. In each experiment, the exciting laser intensity, background level, contrast, and electronic zoom were maintained at the same level.

### Bacterial Surface Hydrophobicity Assay

*Streptococcus mutans* surface hydrophobicity was determined by microbial adhesion to hydrocarbon (Rosenberg, 2006). Briefly, *S. mutans* was adjusted to an optical density (OD_600__nm_) of approximately 0.5. After incubation at 37°C for 0 or 30 min with same concentrations (0, 125, 250, and 500 μg/mL) of cinnamaldehyde as above under an aerobic condition, the tubes were centrifuged at 5000 × *g* for 5 min at 4°C, washed twice with sterile PBS, and resuspended in the same buffer. Absorbance was measured at 550 nm (recorded as OD_1_). Then, the tube was vigorously shaken after adding 20% (v/v) xylene. The mixture was left to settle until the aqueous phase separated from the organic phase. Absorbance of the aqueous phase was measured at 550 nm (recorded as OD_2_). The percent hydrophobicity was calculated by the following equation: H = (OD_1_−OD_2_)/OD_2_ × 100%. The final hydrophobicity was determined using the difference between values at 0 min (H_1_) and 30 min (H_2_). Bacterial surface hydrophobicity assays were performed in triplicate independent.

### Bacterial Aggregation Assay

Aggregation experiments were performed as previously described with minor modifications (Xu et al., 2012). Briefly, an overnight of *S. mutans* suspension was harvested by centrifugation at 12,000 × *g* for 30 s, washed twice with PBS, and resuspended in PBS to an optical density (OD_600__nm_) of approximately 0.5, determined by using a spectrophotometer. The initial OD_600__nm_ was recorded and the samples with same concentrations (0, 125, 250, and 500 μg/mL) of cinnamaldehyde as above were incubated at 37°C for 2 h. The percentage of aggregation was calculated by the following equation: Aggregation rate = (OD_Initial_−OD_2 h_)/(OD_Initial_−OD_Blank_) × 100%. Bacterial aggregation assays were performed in triplicate independent.

### Glycolytic pH Drop

The effect of cinnamaldehyde on *S. mutans* glycolysis was measured as described elsewhere (Belli and Marquis, 1991). Briefly, *S. mutans* was harvested at mid-logarithmic phase, washed with a salt solution (50 mM KCl + 1 mM MgCl_2_), and resuspended in the same salt solution with same concentrations of cinnamaldehyde (0, 125, 250, and 500 μg/mL) as above. Glucose was added to a final concentration of 1% (w/v) and the initial pH of the mixtures was then adjusted to 7.2–7.4 with 0.2 M KOH. The decrease in pH by glycolytic activity of *S. mutans* was monitored at 10 min intervals over a period of 120 min. The experiments were repeated for three times independently.

### Acid Tolerance Assay

The role of cinnamaldehyde on the acid tolerance of *S. mutans* was evaluated by measuring the viability of bacteria after 120 min exposure at pH 5.0 (Svensäter et al., 1997). *S. mutans* were harvested at the mid-logarithmic phase and collected by centrifugation. The cells were resuspended in TYEG (containing 10% tryptone, 5% yeast extract, 3% K_2_HPO_4_, and 1% glucose) medium buffered with 40 mM phosphate-citrate buffer (pH 5.0) with same concentrations (0, 125, 250, and 500 μg/mL) of cinnamaldehyde as above, and incubated at 37°C for 2 h. Samples were removed for viable counts. We counted the number of colony on plates, expressed as CFU/mL after diluting the sample. The number of colonies was calculated following log-transformation, to normalize the data. The experiments were repeated for three times independently.

### RNA Isolation, Reverse Transcription, and Quantitative Real-Time PCR

To evaluate the effect of cinnamaldehyde on the expression of virulence genes of *S. mutans*, the 24 h biofilms formed with different concentrations of cinnamaldehyde (0, 250, and 500 μg/mL) were harvested, resuspended in TRIzol reagent (Sigma-Aldrich). Total RNA extractions were performed according to the manufacturer’s instructions. Purified RNA was dissolved in 20 μL of DEPC-treated water and stored at −80°C until required for cDNA labeling. A cDNA synthesis kit (Takara, Dalian, China) was used to generate cDNAs. The reverse transcription reaction mixture (20 μL), consisting of 4 μL of 5 × Buffer (containing dNTPs and Mg^2+^), 1 μL of PrimeScript RT EnzymeMix I, 1 μL of Oligo (dT) primer (50 μM), 1 μL of random hexamers (100 μM), and 1 μg of RNA sample, was incubated at 37°C for 15 min and the reaction was terminated at 85°C for 5 s, according to the manufacturer’s instructions. The cDNA samples were stored at −20°C until used.

The real-time PCR reaction mixture (20 μL) contained SYBRGreen PCR Master Mix (Takara), 5 μL of template cDNA, and 0.5 μM appropriate forward and reverse PCR primers. The PCR conditions included an initial denaturation at 95°C for 5 min, followed by 40 cycles of denaturation at 95°C for 15 s, annealing and extension at 55°C for 15 s. The resulting cDNA and negative control were amplified using an Applied Biosystems 7900HT Fast Real-Time PCR System (Applied Biosystems). The primer sequences in this study are listed in Table 2. The expression levels of all the tested genes (Table 2) were normalized using the 16S rRNA gene of *S. mutans* (GenBank accession No. X58303) as an internal standard. The cycle threshold (*C*_t_) values are defined as the cycle in which fluorescence becomes detectable above the background fluorescence, and is inversely proportional to the logarithm of the initial number of template molecules. The fold changes in gene expression were using the ΔΔ*C*_t_ method. Each experiment was performed with triplicate samples at each time point. The results correspond to three experiments independently.

**TABLE 2:** Nucleotide sequences of primers used in this study.

			**Amplicon**
**Gene^∗^**	**Description**	**Primer sequence(5′-3′)**	**size (bp)**
*16S rRNA*	Normalizing internal standard	F: CCTACGGGAGGCAGCAGTAG R: CAACAGAGCTTTACGATCCGAAA	100
*gtfB*	Water insoluble glucan production	F: AGCAATGCAGCCAATCTACAAAT R: ACGAACTTTGCCGTTATTGTCA	95
*gtfC*	Water soluble and insoluble glucan production	F: GTGCGCTACACCAATGACAGAG R: GCCTACTGGAACCCAAACACCTA	107
*gtfD*	Water soluble glucan production	F: TGGCACCGCAATATGTCTCTTC R: CAATCCGCAATAACCTGAATACCG	183
*gbpB*	Glucan binding protein	F: ATGGCGGTTATGGACACGTT R: TTTGGCCACCTTGAACACCT	50
*vicR*	Response regulator	F: TGACACGATTACAGCCTTTGATG R: CGTCTAGTTCTGGTAACATTAAGTCCAATA	100
*ciaH*	Response regulator	F: CGTCATCAATAATGTCAATGCCTTC R: TACCTTAACTGTCACTGTCCGATAC	138
*comDE*	Competence-stimulating peptide	F: ACAATTCCTTGAGTTCCATCCAAG R: TGGTCTGCTGCCTGTTGC	80
*ldh*	Lactate dehydrogenase	F: ACTTCACTTGATACTGCTCGTT R: AACACCAGCTACATTGGCATGA	140
*relA*	Guanosine tetra (penta)-phosphate synthetase	F: ACAAAAAGGGTATCGTCCGTACAT R: AATCACGCTTGGTATTGCTAATTG	100

*^∗^Based on the NCBI *S. mutans* genome database.*

### Statistical Analysis

All data are expressed as mean ± standard deviation. One-way analysis of variance (ANOVA) with *Dunnett*’*s post hoc* test was used to calculate the significance of the difference between the biofilms formed by *S. mutans* with or without cinnamaldehyde under the tested conditions (SPSS 15.0 software, United States). *P* < 0.05 was considered statistically significant.

## Results

### Antibacterial Activity of Cinnamaldehyde on Planktonic *S. mutans*

**FIGURE 1:**
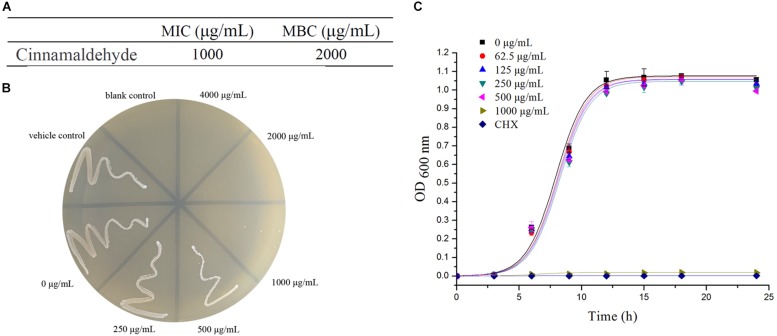
Antibacterial Activity of Cinnamaldehyde. **(A)** MIC and MBC values of cinnamaldehyde against planktonic *S. mutans*. **(B)** The number of colonies on BHI agar. **(C)** Growth curve of planktonic *S. mutans* with different concentrations of cinnamaldehyde. ^∗^*P* < 0.05, ^∗∗^*P* < 0.01, significantly different from the control group.

The MIC and the MBC of cinnamaldehyde against planktonic *S. mutans* were 1000 and 2000 μg/mL, respectively (Figures 1A,B). The results of growth curve assay confirmed that cinnamaldehyde significantly inhibited the growth of *S. mutans* at the concentration of 1000 μg/mL. Compared to the control group, there was no significant alteration in the growth curve of *S. mutans* with cinnamaldehyde under 500 μg/mL of concentration as shown in Figure 1C.

### Cinnamaldehyde Suppresses Biofilm Biomass and Metabolic Activity

**FIGURE 2:**
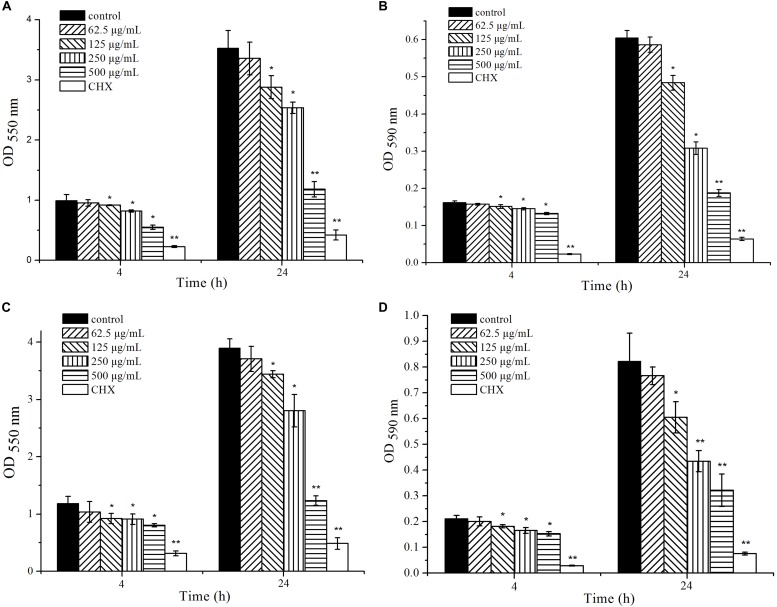
Effect of cinnamaldehyde on overall biomass and metabolic activity of single-species and dual-species biofilm formation at 4 and 24 h time points. **(A)** overall biomass of *S. mutans* biofilm **(B)** metabolic activity of *S. mutans* biofilm **(C)** overall biomass of *S. mutans* and *S. sanguinis* biofilm **(D)** metabolic activity of *S. mutans* and *S. sanguinis* biofilm. ^∗^*P* < 0.05, ^∗∗^*P* < 0.01, significantly different from the control group at 4 and 24 h time points.

The overall biomass of biofilms was quantified by the crystal violet (CV) assay at the concentration ranging up to 500 μg/mL of cinnamaldehyde. There were significant differences in the overall biomass of biofilms at both 4 h and 24 h time points for different concentrations of cinnamaldehyde (except for 62.5 μg/mL) compared with the control group (Figure 2A). At 4 h, *S. mutans* exhibited OD_550__nm_ values of 0.995 ± 0.099. In the presence of cinnamaldehyde, the OD_550__nm_ values after 4 h incubation were 0.917 ± 0.007 at 125 μg/mL, 0.820 ± 0.018 at 250 μg/mL, and 0.551 ± 0.036 at 500 μg/mL concentration. Similar trends were observed after 24 h of incubation. With increasing concentrations of cinnamaldehyde, the biomass of biofilms exhibited OD_550__nm_ values that decreased from 2.879 ± 0.093 at 125 μg/mL to 1.182 ± 0.127 at 500 μg/mL. The biomass of dual-species biofilm was also inhibited by cinnamaldehyde as shown in Figure 2C. The manner of inhibition was similar to that of the *S. mutans* single-species biofilm.

The metabolic activity of biofilms was quantified by the MTT assay with the same concentrations of cinnamaldehyde as mentioned above. The results showed that cinnamaldehyde decreased the biofilm metabolism at both time points compared to the control group and also confirmed the crystal violet assay data (Figure 2B). The metabolic activity of biofilms exhibited OD_590__nm_ values of 0.162 ± 0.004 and 0.605 ± 0.019 at 4 and 24 h, respectively, whereas in the presence of cinnamaldehyde, the values decreased from 0.151 ± 0.003 at 125 μg/mL to 0.125 ± 0.003 at 500 μg/mL after 4 h of incubation and decreased from 0.484 ± 0.020 at 125 μg/mL to 0.188 ± 0.010 at 500 μg/mL after 24 h of incubation. Cinnamaldehyde also inhibited the metabolic activity of dual-species biofilm in a manner similar to its inhibition of the *S. mutans* single-species biofilm (Figure 2D). These results demonstrated that cinnamaldehyde at the concentrations ranging from 125 to 500 μg/mL can effectively reduce biofilm biomass and metabolic activity of *S. mutans* single and dual-species biofilms at different incubation time points during biofilm formation.

### Confocal Microscopic Observation of Biofilm

**FIGURE 3:**
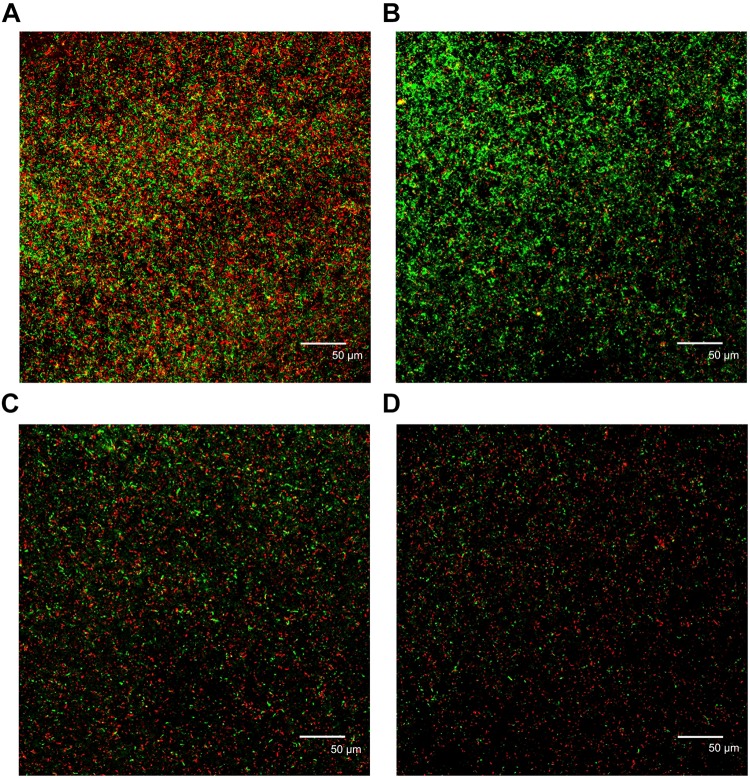
Confocal laser scanning micrographs of biofilms with different concentrations of cinnamaldehyde. **(A)** Control; **(B)** 125 μg/mL; **(C)** 250 μg/mL; **(D)** 500 μg/mL. Red, non-viable cells; green, viable cells; yellow, overlap of non-viable and viable cells. Bar = 50 μm.

The biofilm images formed with different concentrations of cinnamaldehyde after 24 h of incubation were observed using a confocal laser scanning microscope (Figure 3). The images reflect different green (live cells) and red (dead cells) fluorescence intensities. In the absence of cinnamaldehyde, the biofilm had a uniform distribution with a relatively dense structure and complete coverage of the surface (Figure 3A). Following treatment with cinnamaldehyde, the biofilms were highly dispersed and visibly loose (Figures 3B–D). Cinnamaldehyde decreased the surface area visibly covered by the biofilm, which consequently led to significant reduction in the biofilm biomass.

### Cinnamaldehyde Increased Surface Hydrophobicity, Reduced Aggregation

**FIGURE 4:**
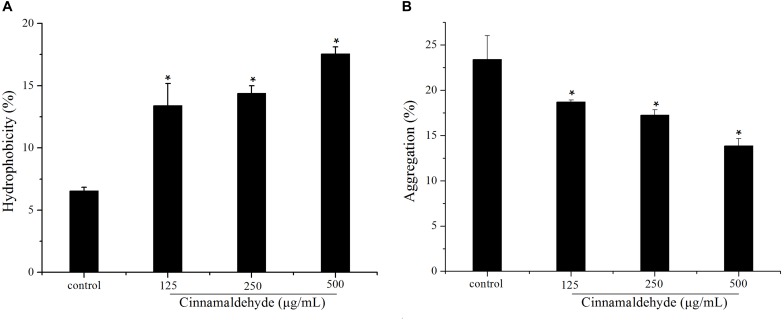
Effect of cinnamaldehyde on hydrophobicity **(A)** and aggregation **(B)** of S. mutans.*P < 0.05, significantly different from the control group.

The hydrophobicity of the bacterial surfaces was determined by measuring the percentage of their adherence to hydrocarbons. Cinnamaldehyde increased *S. mutans* surface hydrophobicity as shown in Figure 4A. The *S. mutans* surface hydrophobicity rates with different concentrations of cinnamaldehyde (125, 250, and 500 μg/mL) were 13.40 ± 1.77%, 14.39 ± 0.62% and 17.56 ± 0.56%, respectively; which were substantially higher than that of the control group (6.54 ± 0.29%). As shown in Figure 4B, the aggregation rate of *S. mutans* reached 23.39 ± 2.61% after 2 h incubation. A dose-dependent decrease in bacterial aggregation was observed with different concentrations of cinnamaldehyde. These results indicated that cinnamaldehyde plays an important role in hydrophobicity and aggregation.

### Cinnamaldehyde Inhibit the Acidogenicity and Acidurity

**FIGURE 5:**
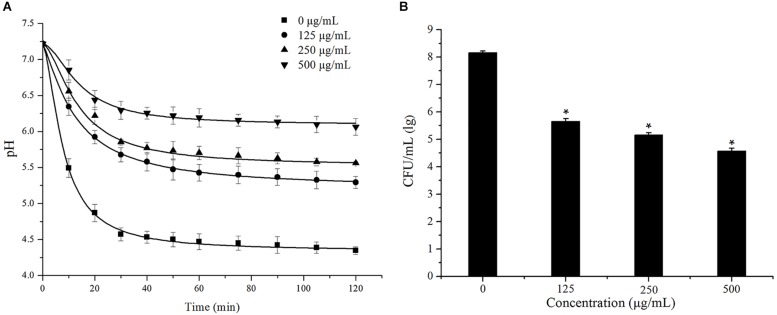
Effect of cinnamaldehyde on acid production **(A)** and acid tolerance **(B)** of S. mutans.*P < 0.05, significantly different from the control group.

The effects of cinnamaldehyde on acid production by *S. mutans* were evaluated using glycolytic pH drop assay. *S. mutans* was cultured in the presence of various concentrations of cinnamaldehyde, and the pH change was measured. As shown in Figure 5A, the pH decreased from 7.21 ± 0.01 to 4.35 ± 0.05 after 120 min of incubation in the control group. The terminal pH increased this acidic pH (4.35 ± 0.05) to 5.29 ± 0.09, 5.56 ± 0.02, 6.06 ± 0.11 after treatment with cinnamaldehyde (125, 250, and 500 μg/mL). The maximum initial pH drop recorded within first 10 min of incubation was observed in the control group maximum (from 7.21 ± 0.01 to 5.49 ± 0.13). However, 500 μg/mL cinnamaldehyde showed minimum initial pH drop from 7.21 ± 0.01 to 6.85 ± 0.14.

The effects of cinnamaldehyde on acid tolerance of *S. mutans* were evaluated and the results are shown in Figure 5B. Compared with the control group, fewer bacterial colonies were formed after treatment with cinnamaldehyde. The survival rates of *S. mutans* at pH 5.0 were significantly reduced in the presence of cinnamaldehyde (125 μg/mL, 250 μg/mL, 500 μg/mL, *P* < 0.05). These results indicated that cinnamaldehyde inhibits *S. mutans* acidogenicity and acidurity.

### Gene Expressions Were Down-Regulated by Cinnamaldehyde Treatment

**FIGURE 6:**
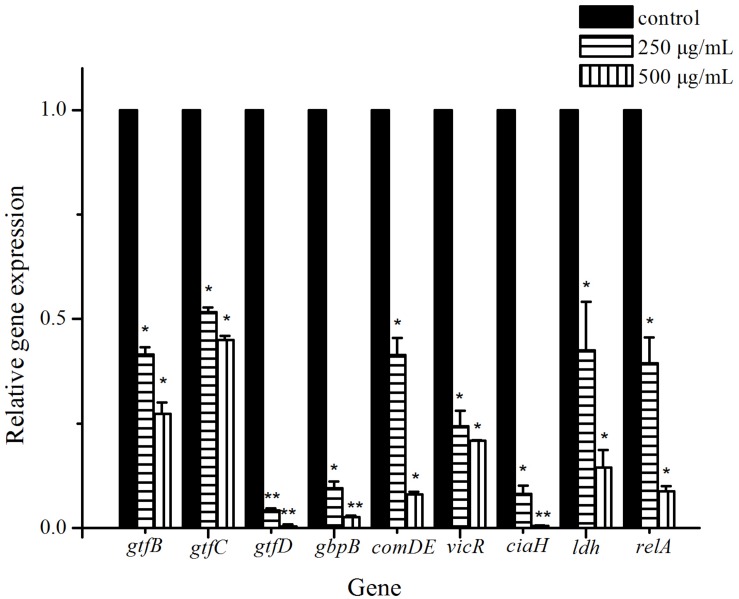
Effect of cinnamaldehyde on gene expression of *S. mutans* biofilms. The results represent the means and SD of three independent experiments performed in triplicate. ^∗^*P* < 0.05, ^∗∗^*P* < 0.01, significantly different from the control group.

To gain insight into biofilm-related gene expression, real-time PCR analysis was used to quantify the effect of 250 and 500 μg/mL cinnamaldehyde on the biofilms formed by *S. mutans*. Among the studied genes, four genes were found to be involved in extracellular polysaccharide synthesis (*gtfB*, *gtfC*, *gtfD*, and *gbpB*). Three genes were found to be related to two-component signal transduction system (*comDE*, *vicR*, and *ciaH*). And *ldh* and *relA* genes encode lactic acid production and guanosine tetra (penta)-phosphate synthetase/hydrolase, respectively. In general, all tested genes (Table 1) were down-regulated in the biofilms treated with 250 and 500 μg/mL cinnamaldehyde compared to the control group and the relative fold changes of gene transcripts decreased with increasing cinnamaldehyde concentration (Figure 6). After 250 and 500 μg/mL cinnamaldehyde treatment, expression of *gtfD* in *S. mutans* biofilms was significantly decreased by 0.0421-, and 0.00380 fold, respectively. After cinnamaldehyde treatment, expression levels of other genes associated with biofilm formation were reduced in the range from 0.517 to 0.0819 fold at 250 μg/mL and from 0.273 to 0.00483 fold at 500 μg/mL.

## Discussion

Dental caries is the most common oral diseases that results from a dysbiosis of tooth-associated biofilms. In recent years, the use of natural plant products for oral diseases has attracted increasing attention and has been widely studied (Kouidhi et al., 2015). In the present study, we selected the natural product, cinnamaldehyde, for investigating its antimicrobial activities against *S. mutans* biofilm. First, we determined that MIC and MBC of cinnamaldehyde against planktonic *S. mutans* UA159 were 1000 and 2000 μg/mL, which was slightly different from previous studies. The MIC of cinnamaldehyde for *S. mutans* (ATCC 25175) was >500 μM by resazurin staining with BHI broth at 37°C in 5% CO_2_ for 24 h (Polaquini et al., 2017). The MBC value for *S. mutans* (DMS20523) was 1728 μg/mL (= 13 mM) with TSB broth at 37°C for 1 h (Ribeiro et al., 2018). The difference may be caused by the use of different bacterial strains, culture media, conditions, and methods. Our results demonstrated that cinnamaldehyde did not significantly affect the bacterial growth rate at concentrations below 500 μg/mL. We investigated the effect of cinnamaldehyde at sub-MIC levels on the development of dental caries by inhibiting *S. mutans* biofilm formation.

Biofilm formation can be divided into several processes, including the initial adherence to a solid surface, reversible attachment to that surface, production of extracellular polymeric substance (EPS), irreversible attachment, and maturation into a complex three-dimensional architecture. In our study, the CV and MTT assays showed that cinnamaldehyde attenuated the biofilm formation at initial adherence stage (4 h) and maturation stage (24 h) more effectively with increasing concentration increasing, ranging from 125 to 500 μg/mL. CV assay was used for quantification of biofilm biomass, while the MTT assay was utilized to evaluate the metabolic activities of viable bacteria in biofilms. Importantly, cinnamaldehyde not only inhibited biofilm formation of *S. mutans* in a single-species model, but also inhibited dual-species biofilm by *S. mutans* and *S. sanguinis* in similar trend. *S. sanguinis* is an early colonizer of tooth surfaces and forms biofilms with other species of microorganisms. *S. mutans* and *S. sanguinis* are predominant members in the dental plaque, which is often described as a dual-species biofilm model (Liu et al., 2011; Pereira et al., 2013; Li et al., 2014; Yoshida et al., 2014; Guo et al., 2019). Confocal imaging also confirmed that the biofilms were highly dispersed and visibly loose upon cinnamaldehyde treatment. These results suggested that unlike antibiotics, the biofilm decrease induced by cinnamaldehyde is not related to planktonic bacterial growth inhibition.

Bacterial properties, such as cell surface hydrophobicity and aggregation are important for adherence to the tooth surface, which is essential for dental biofilm formation and subsequent dental caries (Gibbons and van Houte, 1973; Matsumoto-Nakano et al., 2011). The adhesion of *S. mutans* includes a sucrose-independent mode, which primarily depends on hydrogen bonding and hydrophobic interactions between bacteria and the adhering surface (Hasan et al., 2015). Aggregation is a process through which a strain within the biofilm produces polymers to boost the integration of genetically identical strains (Nyenje et al., 2012). In this study, cinnamaldehyde increased the hydrophobicity and decreased the aggregation of *S. mutans*, which suggests that cinnamaldehyde might inhibit bacteria to adhere on a tooth surface, thereby reducing the biofilm formation, and this finding was consistent with previous studies (Yue et al., 2018).

The ability to produce acid (acidogenicity) and to tolerance to low pH (acidurance) are key physiological factors of *S. mutans* for the demineralization of the tooth surface and formation of dental caries of *S. mutans* (Kuramitsu, 1993; Banas, 2004). We investigated the effect of sub-MIC concentrations of cinnamaldehyde on acid production using a glycolysis pH drop assay. Glycolysis is the main pathway for acid production. The bacteria carry out glycolysis continuously by metabolizing a wide range of dietary carbohydrates, and finally form acids. The critical pH value of 5.0–5.5 is important for the balance between demineralization and remineralization of tooth enamel. If the surrounding solution pH caused by accumulation of acid is less than the critical pH, tooth demineralization and subsequent initiation of dental caries occur (Pandit et al., 2013). Our results showed that with increasing concentrations of cinnamaldehyde, the initial rate of the pH drop gradually reduced and the final pH values were higher than the critical pH value. These findings suggest that cinnamaldehyde impairs in acidogenicity and prevents tooth demineralization, which may be due to the inhibition of the glycolytic enzymes for acid production. Acid tolerance is another main physiological factor associated with the cariogenic potential. Our result indicates that cinnamaldehyde decreases the survival rate of bacteria at pH 5.0. Additionally, the final pH values in the glycolytic pH-drop assay also reflect acid tolerance (Gregoire et al., 2007). Therefore, it is apparent that cinnamaldehyde has a notable effect on the acid production and acid tolerance.

Finally, real-time PCR analysis was performed to evaluate the effect of cinnamaldehyde on the gene expressions of virulence factors in *S. mutans*. Differences in the expression of the various virulence genes provided information on their function in biofilm formation and helped in understanding the process. Our results showed that the expressions of all selected virulence genes were downregulated in the presence of cinnamaldehyde. Among them, GTFase synthesizes glucans which provide binding sites for bacterial adherence, biofilm formation and the development of caries. *S. mutans* has at least three GTF enzymes (GTFB, GTFC, and GTFD), according to the types of glucans they synthesize. GTFB (encoded by *gtfB*) synthesizes water-insoluble polysaccharide containing α-1,3-linked glucans, which contributes to the scaffolding of the EPS matrix and facilitates cell aggregation in stable biofilms. GTFC (encoded by *gtfC*) catalyzes the synthesis of a mixture of water insoluble and alkali soluble glucan from sucrose, with both α-1,3 and α-1,6-linked glucans, which are required for plaque formation and structurally stable biofilms. GTFD (encoded by *gtfD*) synthesizes water-soluble glucans containing α-1,6-linked glucans, which allows interaction with salivary proteins in the pellicle (Veloz et al., 2016; De et al., 2018; Gabe et al., 2019). Mutant strains of *S. mutans* defective in *gtf* genes, especially *gtfB* and *gtfC*, are far less cariogenic than the wild type strains *in vivo* (Yamashita et al., 1993). The *gbpB* gene, which encodes surface-associated glucan binding protein (GBPB), has affinity for glucans, mediating oral bacterial cell-cell aggregation, promoting bacterial adhesion and biofilm maturation process (Duque et al., 2011; Fujita et al., 2011). In our study, the reduction of these gene expressions may result in decreased production of extracellular polysaccharide and oral bacterial aggregation, thus inhibiting biofilm formation.

The two-component signal transduction system (TCS) plays important roles in response for multiple environmental stress responses, production of virulence factors, and biofilm formation. *S. mutans* UA159 contains 14 pairs of TCS, including VicRK, CiaRH and ComDE (Lévesque et al., 2007; He et al., 2008). The VicRK system was found to be involved in regulation of sucrose-dependent adhesion, competence development, and biofilm formation. *vicR* gene encodes a VicR response regulator, which is an essential part of VicRKX TCS. It is known to regulate a set of genes coding for synthesis of glucan matrix (*gtfB*, *gtfC*, *gtfD*, *gbpB*), which are critical for adherence to a smooth tooth surface (Senadheera et al., 2005; Viszwapriya et al., 2017). CiaH/CiaR is another major TCSs related to biofilm formation, acid tolerance, and genetic competence. The deletion of the *ciaH* gene affects bacterial attachment, reduced sucrose-independent biofilm formation, abolished mutacin production, and diminished competence development (Tam et al., 2007; Wu et al., 2010). ComDE system, involved in competence regulation as well as bacteriocin production, is also the most common intraspecific cell-cell communication quorum sensing system in *S. mutans*. The quorum sensing system is an essential component of gene regulation networks responsible for the adaptation of bacteria in biofilms and stress responses. It can sense, respond to environmental fluctuations, and regulate a number of physiological activities including biofilm formation. It has a positive regulatory effect on the expression of biofilm-related genes such as *gtfB*, *gtfC* and *gbpB* in *S. mutans*, and inactivation of Com pathway results in biofilm defects (Li et al., 2008; Senadheera and Cvitkovitch, 2008; Kaur et al., 2017). Hence, down-regulation of *comDE* by cinnamaldehyde suppresses quorum sensing mechanism resulting in biofilm inhibition.

Lactate dehydrogenase (LDH, encoded by *ldh* gene) is one of the most important enzymes in the process of glycolysis, which generate lactic acid in *S. mutans*. LDH and lactic acid facilitate *S. mutans* to dissolve tooth minerals and cause dental caries. Strains deficient in LDH display reduced cariogenicity (Fitzgerald et al., 1989; Zhang et al., 2016). *relA* gene encodes guanosine tetra (penta)-phosphate synthetase/hydrolase and is known to be involved in several processes, such as acid and oxidative stress tolerance mechanisms, and biofilm formation (Lemos et al., 2004; Liu et al., 2011). The suppression of these genes will impair acid tolerance and result in decreased virulence expressions.

## Conclusion

In conclusion, our study demonstrated that cinnamaldehyde exhibits antimicrobial activity against *S. mutans* biofilm formation by modulating its hydrophobicity, aggregation, acid production, acid tolerance, and virulence gene expression. Therefore, cinnamaldehyde as a natural plant-derived compound may be useful to influence antimicrobial activity against *S. mutans* biofilm. Considering plaque biofilm is produced through multiple regulatory systems, further studies are required for the better understanding of the molecular mechanism underlying the inhibitory effect of cinnamaldehyde on *S. mutans* biofilm formation.

## Data Availability Statement

All datasets generated for this study are included in the manuscript/supplementary files.

## Author Contributions

WZ designed the project and supervised all the experiments. ZYH performed the experiments. ZYH, ZWH, and WJ analyzed the data and wrote the manuscript. All authors read and revised the manuscript.

## Conflict of Interest

The authors declare that the research was conducted in the absence of any commercial or financial relationships that could be construed as a potential conflict of interest.

## References

[B1] Abreu A. C. Saavedra M. J. Simões L. C. Simões M. (2016). Combinatorial approaches with selected phytochemicals to increase antibiotic efficacy against *Staphylococcus aureus* biofilms. *Biofouling* 32 1103–1114. 10.1080/08927014.2016.1232402 27643487

[B2] Adams T. B. Cohen S. M. Doull J. Feron V. J. Goodman J. I. Marnett L. J. (2004). The FEMA GRAS assessment of cinnamyl derivates used as flavor ingredients. *Food Chem. Toxicol.* 42 157–185. 10.1016/j.fct.2003.08.021 14667463

[B3] Al-Dulaijan Y. A. Cheng L. Weir M. D. Melo M. A. S. Liu H. Oates T. W. (2018). Novel rechargeable calcium phosphate nanocomposite with antibacterial activity to suppress biofilm acids and dental caries. *J. Dent.* 72 44–52. 10.1016/j.jdent.2018.03.003 29526668

[B4] Anderson A. C. Rothballer M. Altenburger M. J. Woelber J. P. Karygianni L. Lagkouvardos I. (2018). In-vivo shift of the microbiota in oral biofilm in response to frequent sucrose consumption. *Sci. Rep.* 8:14202. 10.1038/s41598-018-32544-6 30242260 PMC6155074

[B5] Banas J. A. (2004). Virulence properties of *Streptococcus mutans*. *Front. Biosci.* 9:1267–1277. 10.2741/1305 14977543

[B6] Bedran T. B. Grignon L. Spolidorio D. P. Grenier D. (2014). Subinhibitory concentrations of triclosan promote *Streptococcus mutans* biofilm formation and adherence to oral epithelial cells. *PLoS One* 9:e89059. 10.1371/journal.pone.0089059 24551218 PMC3923858

[B7] Belli W. A. Marquis R. E. (1991). Adaptation of *Streptococcus mutans* and *Enterococcus hirae* to acid stress in continuous culture. *Appl. Environ. Microbiol.* 57 1134–1138. 1829347 10.1128/aem.57.4.1134-1138.1991 PMC182857

[B8] Borges A. Abreu A. C. Dias C. Saavedra M. J. Borges F. Simões M. (2016). New perspectives on the use of phytochemicals as an emergent strategy to control bacterial infections including biofilms. *Molecules* 21:E877. 10.3390/molecules21070877 27399652 PMC6274140

[B9] Cai Y. Liao Y. Brandt B. W. Wei X. Liu H. Crielaard W. (2017). The fitness cost of fluoride resistance for different *Streptococcus mutans* strains in biofilms. *Front. Microbiol.* 8:1630. 10.3389/fmicb.2017.01630 28894441 PMC5581503

[B10] Chakraborty B. Burne R. A. (2017). Effects of arginine on *Streptococcus mutans* growth, virulence gene expression, and stress tolerance. *Appl. Environ. Microbiol.* 83 e496–e417. 10.1128/AEM.00496-17 28526785 PMC5514675

[B11] Clinical and Laboratory Standards Institute (2012). *Performance Standards for Antimicrobial Susceptibility Testing; Twenty-Second Informational Supplement. CLSI Document M100-S22*. Wayne, PA: Clinical and Laboratory Standards Institute.

[B12] De A. Jorgensen A. N. Beatty W. L. Lemos J. Wen Z. T. (2018). Deficiency of MecA in *Streptococcus mutans* causes major defects in cell envelope biogenesis, cell division, and biofilm formation. *Front. Microbiol.* 9:2130. 10.3389/fmicb.2018.02130 30254619 PMC6141683

[B13] Duque C. Stipp R. N. Wang B. Smith D. J. Höfling J. F. Kuramitsu H. K. (2011). Downregulation of GbpB, a component of the VicRK regulon, affects biofilm formation and cell surface characteristics of *Streptococcus mutans*. *Infect. Immun.* 79 786–796. 10.1128/IAI.00725-10 21078847 PMC3028841

[B14] Fitzgerald R. J. Adams B. O. Sandham H. J. Abhyankar S. (1989). Cariogenicity of alactate dehydrogenase-deficient mutant of *Streptococcus mutans* serotype c in gnotobiotic rats. *Infect. Immun.* 57 823–826. 2917788 10.1128/iai.57.3.823-826.1989 PMC313183

[B15] Fujita K. Takashima Y. Inagaki S. Nagayama K. Nomura R. Ardin A. C. (2011). Correlation of biological properties with glucan-binding protein B expression profile in *Streptococcus mutans* clinical isolates. *Arch. Oral Biol.* 56 258–263. 10.1016/j.archoralbio.2010.09.018 20979990

[B16] Gabe V. Kacergius T. Abu-Lafi S. Kalesinskas P. Masalha M. Falah M. (2019). Inhibitory effects of ethyl gallate on *Streptococcus mutans* biofilm formation by optical profilometry and gene expression analysis. *Molecules* 24:E529. 10.3390/molecules24030529 30717122 PMC6384797

[B17] Gibbons R. J. van Houte J. (1973). On the formation of dental plaques. *J. Periodontol.* 44 347–360. 10.1902/jop.1973.44.6.347 4575463

[B18] Gregoire S. Singh A. P. Vorsa N. Koo H. (2007). Influence of cranberry phenolics on glucan synthesis by glucosyltransferases and *Streptococcus mutans* acidogenicity. *J. Appl. Microbiol.* 103 1960–1968. 10.1111/j.1365-2672.2007.03441.x 17953606

[B19] Guo X. Liu S. Zhou X. Hu H. Zhang K. Du X. (2019). Effect of D-cysteine on dual-species biofilms of *Streptococcus mutans* and *Streptococcus sanguinis*. *Sci. Rep.* 9:6689. 10.1038/s41598-019-43081-1 31040318 PMC6491432

[B20] Hasan S. Danishuddin M. Khan A. U. (2015). Inhibitory effect of zingiber officinale towards *Streptococcus mutans* virulence and caries development: in vitro and in vivo studies. *BMC Microbiol.* 15:1. 10.1186/s12866-014-0320-5 25591663 PMC4316655

[B21] He X. Wu C. Yarbrough D. Sim L. Niu G. Merritt J. (2008). The cia operon of *Streptococcus mutans* encodes a unique component required for calcium-mediated autoregulation. *Mol. Microbiol.* 70 112–126. 10.1111/j.1365-2958.2008.06390.x 18681938 PMC2955730

[B22] He Z. Wang Q. Hu Y. Liang J. Jiang Y. Ma R. (2012). Use of the quorum sensing inhibitor furanone C-30 to interfere with biofilm formation by *Streptococcus mutans* and its *luxS* mutant strain. *Int. J. Antimicrob. Agents.* 40 30–35. 10.1016/j.ijantimicag.2012.03.016 22578766

[B23] Hu X. Huang Y. Y. Wang Y. Wang X. Hamblin M. R. (2018a). Antimicrobial photodynamic therapy to control clinically relevant biofilm infections. *Front. Microbiol.* 9:1299. 10.3389/fmicb.2018.01299 29997579 PMC6030385

[B24] Hu X. Wang Y. Gao L. Jiang W. Lin W. Niu C. (2018b). The impairment of methyl metabolism from luxS mutation of *Streptococcus mutans*. *Front. Microbiol.* 9:404. 10.3389/fmicb.2018.00404 29657574 PMC5890193

[B25] Jeon J. G. Rosalen P. L. Falsetta M. L. Koo H. (2011). Natural products in caries research: current (limited) knowledge, challenges and future perspective. *Caries Res.* 45 243–263. 10.1159/000327250 21576957 PMC3104868

[B26] Kaur G. Balamurugan P. Princy S. A. (2017). Inhibition of the quorum sensing system (ComDE Pathway) by aromatic 1,3-di-m-tolylurea (DMTU): cariostatic effect with fluoride in wistar rats. *Front. Cell. Infect. Microbiol.* 7:313. 10.3389/fcimb.2017.00313 28748175 PMC5506180

[B27] Kouidhi B. Al Qurashi Y. M. Chaieb K. (2015). Drug resistance of bacterial dental biofilm and the potential use of natural compounds as alternative for prevention and treatment. *Microb. Pathog.* 80 39–49. 10.1016/j.micpath.2015.02.007 25708507

[B28] Kuang X. Chen V. Xu X. (2018). Novel approaches to the control of oral microbial biofilms. *Biomed. Res. Int.* 2018:6498932. 10.1155/2018/6498932 30687755 PMC6330817

[B29] Kuramitsu H. K. (1993). Virulence factors of mutans streptococci: role of molecular genetics. *Crit. Rev. Oral Biol. Med.* 4 159–176. 10.1177/10454411930040020201 8435464

[B30] Lee R. Balick M. J. (2005). Sweet wood—cinnamon and its importance as a spice and medicine. *Explore* 1 61–64. 10.1016/j.explore.2004.10.011 16781503

[B31] Lemos J. A. Brown T. A. Jr. Burne R. A. (2004). Effects of RelA on key virulence properties of planktonic and biofilm populations of *Streptococcus mutans*. *Infect. Immun.* 72 1431–1440. 10.1128/iai.72.3.1431-1440.2004 14977948 PMC356000

[B32] Lévesque C. M. Mair R. W. Perry J. A. Lau P. C. Li Y. H. Cvitkovitch D. G. (2007). Systemic inactivation and phenotypic characterization of two-component systems in expression of *Streptococcus mutans* virulence properties. *Lett. Appl. Microbiol.* 45 398–404. 10.1111/j.1472-765X.2007.02203.x 17897382 PMC2062497

[B33] Li M. Huang R. Zhou X. Zhang K. Zheng X. Gregory R. L. (2014). Effect of nicotine on dual-species biofilms of *Streptococcus mutans* and *Streptococcus sanguinis*. *FEMS Microbiol. Lett.* 350 125–132. 10.1111/1574-6968.12317 24164376

[B34] Li Y. H. Tian X. L. Layton G. Norgaard C. Sisson G. (2008). Additive attenuation of virulence and cariogenic potential of *Streptococcus mutans* by simultaneous inactivation of the ComCDE quorum-sensing system and HK/RR11 two component regulatory system. *Microbiology* 154 3256–3265. 10.1099/mic.0.2008/019455-0 18957580

[B35] Liu B. H. Yu L. C. (2017). In-situ, time-lapse study of extracellular polymeric substance discharge in *Streptococcus mutans* biofilm. *Colloids Surf. B Biointerfaces* 150 98–105. 10.1016/j.colsurfb.2016.11.031 27907861

[B36] Liu C. Worthington R. J. Melander C. Wu H. (2011). A new small molecule specifically inhibits the cariogenic bacterium *Streptococcus mutans* in multispecies biofilms. *Antimicrob. Agents. Chemother.* 55 2679–2687. 10.1128/AAC.01496-10 21402858 PMC3101470

[B37] Martins M. L. Leite K. L. F. Pacheco-Filho E. F. Pereira A. F. M. Romanos M. T. V. Maia L. C. (2018). Efficacy of red propolis hydro-alcoholic extract in controlling *Streptococcus mutans* biofilm build-up and dental enamel demineralization. *Arch. Oral Biol.* 93 56–65. 10.1016/j.archoralbio.2018.05.017 29807235

[B38] Matsumoto-Nakano M. Nagayama K. Kitagori H. Fujita K. Inagaki S. Takashima Y. (2011). Inhibitory Effects of *Oenothera biennis* (Evening Primrose) seed extract on *Streptococcus mutans* and *S.* mutans-induced dental caries in rats. *Caries Res.* 45 56–63. 10.1159/000323376 21311187

[B39] Nyenje M. E. Green E. Ndip R. N. (2012). Biofilm formation and adherence characteristics of *Listeria ivanovii* strains isolated from ready-to-eat foods in alice, South Africa. *Sci. World J.* 2012:873909. 10.1100/2012/873909 23365535 PMC3541635

[B40] Nyvad B. Crielaard W. Mira A. Takahashi N. Beighton D. (2013). Dental caries from a molecular microbiological perspective. *Caries Res.* 47 89–102. 10.1159/000345367 23207320

[B41] Ong K. S. Mawang C. I. Daniel-Jambun D. Lim Y. Y. Lee S. M. (2018). Current anti-biofilm strategies and potential of antioxidants in biofilm control. *Expert Rev. Anti. Infect. Ther.* 16 855–864. 10.1080/14787210.2018.1535898 30308132

[B42] Pandit S. Chang K. W. Jeon J. G. (2013). Effects of *Withania somnifera* on the growth and virulence properties of *Streptococcus mutans* and *Streptococcus sobrinus* at sub-MIC levels. *Anaerobe* 19 1–8. 10.1016/j.anaerobe.2012.10.007 23142795

[B43] Pereira C. A. Costa A. C. Carreira C. M. Junqueira J. C. Jorge A. O. (2013). Photodynamic inactivation of *Streptococcus mutans* and *Streptococcus sanguinis* biofilms in vitro. *Lasers Med. Sci.* 28 859–864. 10.1007/s10103-012-1175-3 22847685

[B44] Polaquini C. R. Torrezan G. S. Santos V. R. Nazaré A. C. Campos D. L. Almeida L. A. (2017). Antibacterial and antitubercular activities of cinnamylideneacetophenones. *Molecules* 22:E1685. 10.3390/molecules22101685 28994740 PMC6151560

[B45] Ren Z. Chen L. Li J. Li Y. (2016). Inhibition of *Streptococcus mutans* polysaccharide synthesis by molecules targeting glycosyltransferase activity. *J. Oral. Microbiol.* 8:31095. 10.3402/jom.v8.31095 27105419 PMC4841093

[B46] Ribeiro M. Malheiro J. Grenho L. Fernandes M. H. Simões M. (2018). Cytotoxicity and antimicrobial action of selected phytochemicals against planktonic and sessile *Streptococcus mutans*. *PeerJ* 6:e4872. 10.7717/peerj.4872 29888127 PMC5991298

[B47] Rosenberg M. (2006). Microbial adhesion to hydrocarbons: twenty-five years of doing MATH. *FEMS Microbiol. Lett.* 262 129–134. 10.1111/j.1574-6968.2006.00291.x 16923066

[B48] Senadheera D. Cvitkovitch D. G. (2008). Quorum sensing and biofilm formation by *Streptococcus mutans*. *Adv. Exp. Med. Biol.* 631 178–188. 10.1007/978-0-387-78885-2_12 18792689

[B49] Senadheera M. D. Guggenheim B. Spatafora G. A. Huang Y. C. Choi J. Hung D. C. (2005). A VicRK signal transduction system in *Streptococcus mutans* affects gtfBCD, gbpB, and ftf expression, biofilm formation, and genetic competence development. *J. Bacteriol.* 187 4064–4076. 10.1128/jb.187.12.4064-4076.2005 15937169 PMC1151735

[B50] Svensäter G. Larsson U. B. Greif E. C. Cvitkovitch D. G. Hamilton I. R. (1997). Acid tolerance response and survival byoral bacteria. *Oral Microbiol. Immun.* 12 266–273. 10.1111/j.1399-302x.1997.tb00390.x 9467379

[B51] Tam K. Kinsinger N. Ayala P. Qi F. Shi W. Myung N. V. (2007). Real-Time monitoring of *Streptococcus mutans* biofilm formation using a quartz crystal microbalance. *Caries Res.* 41 474–483. 10.1159/000108321 17851235 PMC2820325

[B52] Topa S. H. Subramoni S. Palombo E. A. Kingshott P. Rice S. A. Blackall L. L. (2018). Cinnamaldehyde disrupts biofilm formation and swarming motility of *Pseudomonas aeruginosa*. *Microbiology* 164 1087–1097. 10.1099/mic.0.000692 29993359

[B53] Veloz J. J. Saavedra N. Alvear M. Zambrano T. Barrientos L. Salazar L. A. (2016). Polyphenol-rich extract from propolis reduces the expression and activity of *Streptococcus mutans* glucosyltransferases at subinhibitory concentrations. *Biomed. Res. Int.* 2016:e4302706. 10.1155/2016/4302706 27110563 PMC4821976

[B54] Viszwapriya D. Subramenium G. A. Radhika S. Pandian S. K. (2017). Betulin inhibits cariogenic properties of *Streptococcus mutans* by targeting vicRK and gtf genes. *Antonie Van Leeuwenhoek* 110 153–165. 10.1007/s10482-016-0785-3 27757704

[B55] Wu C. Ayala E. A. Downey J. S. Merritt J. Goodman S. D. Qi F. (2010). Regulation of ciaXRH operon expression and identification of the CiaR regulon in *Streptococcus mutans*. *J. Bacteriol.* 192 4669–4679. 10.1128/JB.00556-10 20639331 PMC2937423

[B56] Xu X. Zhou X. D. Wu C. D. (2012). Tea catechin epigallocatechin gallate inhibits *Streptococcus mutans* biofilm formation by suppressing *gtf* genes. *Arch. Oral Biol.* 57 678–683. 10.1016/j.archoralbio.2011.10.021 22169220

[B57] Yamashita Y. Bowen W. H. Burne R. A. Kuramitsu H. K. (1993). Role of the *Streptococcus mutans* gtf genes in caries induction in the specific-pathogen-free rat model. *Infect. Immun.* 61 3811–3817. 8359902 10.1128/iai.61.9.3811-3817.1993 PMC281081

[B58] Ye W. H. Fan B. Purcell W. Meghil M. M. Cutler C. W. Bergeron B. E. (2019). Anti-biofilm efficacy of root canal irrigants against in-situ *Enterococcus faecalis* biofilms in root canals, isthmuses and dentinal tubules. *J. Dent.* 79 68–76. 10.1016/j.jdent.2018.10.002 30296552

[B59] Yoshida Y. Konno H. Nagano K. Abiko Y. Nakamura Y. Tanaka Y. (2014). The influence of a glucosyltransferase, encoded by gtfP, on biofilm formation by *Streptococcus sanguinis* in a dual-species model. *APMIS* 122 951–960. 10.1111/apm.12238 24628454

[B60] Yue J. Yang H. Liu S. Song F. Guo J. Huang C. (2018). Influence of naringenin on the biofilm formation of *Streptococcus mutans*. *J. Dent.* 76 24–31. 10.1016/j.jdent.2018.04.013 29679633

[B61] Zhang H. Zhou W. Zhang W. Yang A. Liu Y. Jiang Y. (2014). Inhibitory effects of citral, cinnamaldehyde, and tea polyphenols on mixed biofilm formation by foodborne *Staphylococcus aureus* and *Salmonella Enteritidis*. *J. Food Prot.* 77 927–933. 10.4315/0362-028X.JFP-13-497 24853514

[B62] Zhang J. Liu J. Ling J. Tong Z. Fu Y. Liang M. (2016). Inactivation of glutamate racemase (MurI) eliminates virulence in *Streptococcus mutans*. *Microbiol. Res.* 18 1–8. 10.1016/j.micres.2016.02.003 27242137

